# Transurethral Incision of the Bladder Neck at Three Points with a Needle-Type Electrode for Bladder Neck Contracture

**DOI:** 10.3389/fsurg.2022.871099

**Published:** 2022-05-09

**Authors:** Guihua Cao, Liangcheng Liu, Jianping Du, Wei Li, Qiang Li, Na Luo, Xun Liu, Junjie Zhou, Tao Wu

**Affiliations:** ^1^ Department of Urology, People’s Hospital of Leshan, Leshan, China; ^2^ Department of Pathology, People’s Hospital of Leshan, Leshan, China; ^3^ Department of Urology, Affiliated Hospital of North Sichuan Medical College, Nanchong, China

**Keywords:** bladder neck contracture, transurethral incision of the bladder neck, needle-type electrode, benign prostatic hyperplasia, transurethral endoscopic treatment

## Abstract

**Purpose:**

This study aims to evaluate the efficacy of transurethral incision of the bladder neck (TUIBN) at three points with a needle-type electrode for treatment of bladder neck contracture (BNC).

**Materials and Methods:**

Between January 2016 and April 2021, the bladder necks of 53 patients with BNC after surgery were incised by the needle-type electrode at the 5, 7, and 12 O’clock positions. Patient’s preoperational characteristics, peri- and postsurgical outcomes, such as time of operation, postoperative bladder irrigation, and postoperative hospital stay, and data of the international prostate symptom score (IPSS), maximum flow rate (Qmax), and postvoid residual (PVR) were recorded 3 and 6 months after surgery.

**Results:**

All 53 cases of BNC were successfully treated in 35.00 (25.00, 45.00) min with 18.00 (14.00, 21.00) h for postoperative bladder irrigation with little intraoperative bleeding (less than 50 mL). The postoperative hospital stay ranged from 2 to 8 days, a mean of 3.50 (3.00, 5.00) days. No major intraoperative or postoperative complications were observed. All cases that underwent follow-up assessment at 3 and 6 months after the surgery showed significantly decreased IPSS and PVR and increased Qmax compared to preoperation ones (*p* ≤ 0.001). Of these 53 patients, there was no recurrence in severe BNC patients, but 5 of 53 (9.4%) BNC patients developed BNC again within 6 months and required repeated TUIBN. Thirty patients comprised five recurrent cases with a follow-up period of more than 1 year.

**Conclusions:**

TUIBN at three points provides a safe, effective, and reliable option in treating patients with BNC.

## Introduction

Benign prostate hyperplasia (BPH) is a common disease among the elderly (1), which is the major cause of lower urinary tract symptoms. Surgical treatment is often the most effective intervention for such a disease. It has been reported that in transurethral resection of prostate (TURP), different energy laser sources and prostatic tissue ablation, such as photoselective vaporization of the prostate (PVP) with a green light laser, holmium laser enucleation or ablation of the prostate, and thulium vapoenucleation laser, are widely used in the clinical setting (2). However, complications of urethral stricture and BNC occur postoperation, which cause poor quality of life in patients. The reported incidence range of BNC is from 0.3% to 9.6% (3–6). Risk factors of BNC include a low adenoma volume, excessive electrocoagulation, resection of the bladder neck, and long operative time (7–9). Bladder neck incision (BNI), laser vaporization, balloon dilatation, and stent placement are applied for the treatment of BNC with overall high recurrence rates (10).

In recent years, new techniques such as T-plasty, a modified YV-plasty, and robot-assisted laparoscopic YV plasty have been performed in the treatment of BNC and have achieved satisfactory outcomes with a success rate of 83.3%–90% (11, 12). However, transurethral endoscopic treatment is still widely accepted as the initial treatment of BNC because of little trauma, low complications, and quick recovery. The present study aims to perform TUIBN at three points with a needle-type electrode for the treatment of bladder neck contracture and evaluate the efficacy of the procedure.

## Materials and Methods

### Study Population

A total of 53 patients between January 2016 and April 2021 were included in the present study, following approval by The Ethics Committee of the People’s Hospital of Leshan (approval no. LW2022-01-01). These patients participated in this study between 3 and 120 months after TURP, PVP, or suprapubic prostatectomy. The mean time to diagnose BNC was 22.9 months; 20 patients (37.7%) developed BNC within 6 months and 13 (24.5%) within 3 months. All patients were diagnosed with BNC under cystoscopy. The severity of BNC was classified into three grades (13): grade 1, the mild-17F cystoscopic sheath can pass through the bladder neck by force but 22F cystoscopic sheath cannot (diameter of the bladder neck between 5 and 7 mm); grade 2, the moderate-17F cystoscopic sheath cannot pass through the bladder neck (diameter of the bladder neck between 2 and 5 mm); and grade 3, severe pinpoint-like hole (less than 2 mm). There were 24, 19, and 10 patients with mild, moderate, and severe BNC, respectively.

### Assessment Parameters

Patient’s preoperational properties, pre- and postsurgical data, including age, the volume of prostate before the first surgical procedure for the treatment of BPH (the volume of the prostate was assessed by transrectal ultrasonography and obtained as height × length × width × 0.52), previous surgical procedures, weight of excised scar tissue, surgical complications, and time of operation, occurrence of BNC, postoperative bladder irrigation, and postoperative hospital stay, and data of the international prostate symptom score (IPSS), postvoid residual (PVR), and maximum flow rate (Qmax) were recorded 3 and 6 months after TUIBN.

### Instruments and Surgical Procedures

All patients provided informed consent before transurethral surgery. A team of urologists with similar experience performed all transurethral surgeries. Combined spinal and epidural anesthesia was performed on each patient in the lithotomy position. Major equipment and instrument for surgery included the following: an Olympus Plasmakinetic system generator (Olympus, Japan), a needle-type electrode (Olympus, Japan), a resectoscope (Olympus, Japan), a ureteroscope (Wolf, Germany), and a zebra guidewire (Boston Scientific, MA, USA). The generator settings for treating BNC were 240 and 120 W for cutting and coagulation, respectively. Normal saline was used for irrigation.

**Figure 1:**
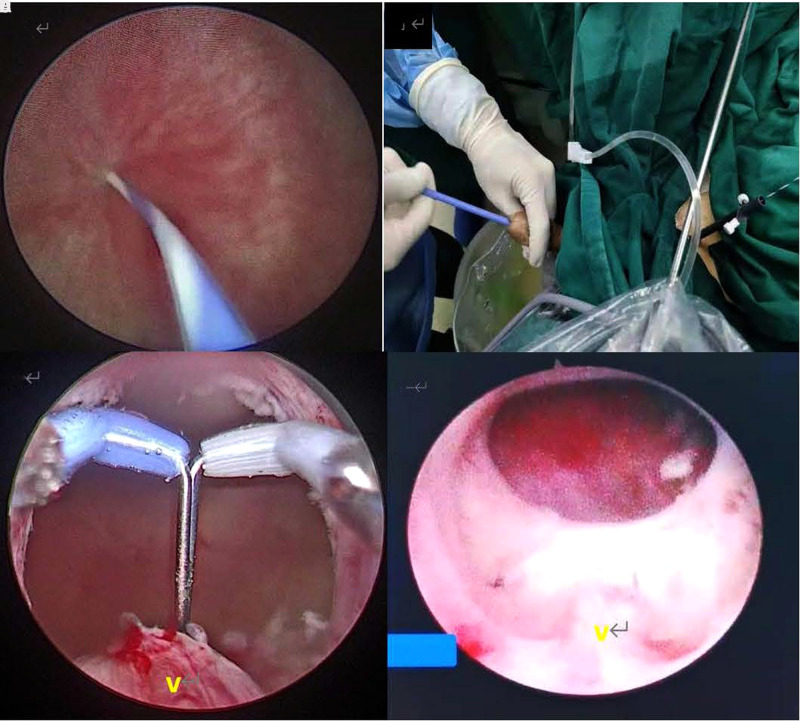
(**A**) Introperative ureteroscopic view of a pinpoint-like bladder neck; (**B**) suprapubic cystostomy and dilation of the bladder neck; (**C**) immediate resectoscopic view of a bladder neck after TUIBN; (**D**) cystoscopic view of a bladder neck 18 months after TUIBN.

**Figure 2:**
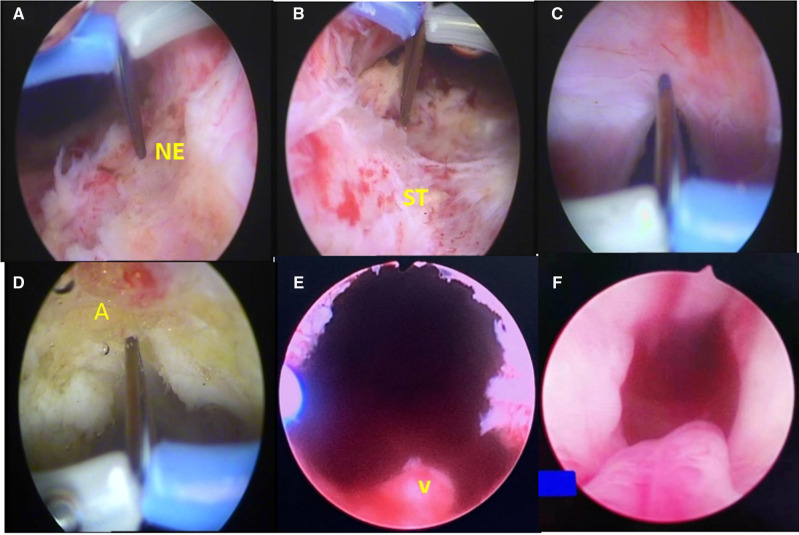
(**A–D**) The incisions were performed at 5, 7, and 12 O’clock positions using a needle electrode (NE) to create an incisional pathway from the bladder neck to the proximal verumontanum (V), which reached the adipose layer (A); (**E**) immediate resectoscopic view of the bladder neck after TUIBN; (**F**) cystoscopic view of the bladder neck 1 month after TUIBN.

Transurethral insertion of the Olympus 26F resectoscope was under direct vision. For a mild or moderate BNC case, a cutting loop was pushed retrogradely to enter the bladder. For the severe BNC case, a zebra guidewire was inserted into the bladder under the direct vision of the ureteroscope through the urethra to determine the right pathway. If failed in the retrograde course, suprapubic cystostomy was performed and the ureteroscope was inserted by the tract of cystostomy to enter the bladder. The zebra guidewire was inserted into the urethra from the bladder neck, which contracted as a pinpoint-like hole. The bladder neck could be expanded along the guidewire by using a dilator (**Figure 1**), and the resectoscope was then inserted along the zebra guidewire to enter the bladder. Following that, the incisions were performed at 5, 7, and 12 O’clock positions using an Olympus needle-type electrode to create an incisional pathway from the bladder neck to the proximal verumontanum, which reached the adipose layer; the bladder neck opened wider and wider as the fibrous rings leading to contracture were completely cut off. Finally, the redundant scar tissues of the bladder neck and prostatic urethra were excised with the cutting loop, making the bladder neck parallel to the vesical trigone (**Figure 2**). The 22F urethral catheter was retained after surgery for continuous irrigation of the bladder (14).

### Statistical Analysis

The normal distribution data were represented by mean ± standard deviation; non-normal distribution data were represented by median and quartile. The comparison of indexes before and after surgery was self-paired (self-paired design, using a paired *t*-test or a paired rank-sum test according to whether the difference was normally distributed); *Z* was the *Z*-value of the paired rank-sum test, *t* was the paired *t*-test value, and *p* < 0.05 was statistically significant based on SPSS25.0 statistical analysis software.

## Results

The patients’ baseline characteristics and perioperative results are shown in **Table 1**. The previous mean prostate volume was 30.05 (24.95, 37.18) mL. Of the 53 patients, 44 underwent TURP once or PVP before the development of BNC, 3 underwent TURP twice, and 6 underwent suprapubic prostatectomy once. All surgeries went smoothly, with the time of TUIBN varying from 15 to 70 min, 35.00 (25.00, 45.00) min. There was little intraoperative bleeding (less than 50 mL). The excised mean weight of redundant scar tissue of the bladder neck and prostatic tissue was 2.10 (0.65, 8.35) g. The mean time of postoperative bladder irrigation was 18.00 (14.00, 21.00) h. The postoperative hospital stay ranged from 2 to 8 days, mean 3.50 (3.00, 5.00) days. During perioperation, no patients had serious bleeding. Three cases of venous sinus hemorrhage were resolved by continuous balloon compression with catheterization. One patient was with hydroabdomen. One case of dysuria following the catheter removal 4 days after surgery, however, resolved completely in the further course of catheterization. Another case of dysuria was due to the presence of a large bladder diverticulum, but there was no recurrent BNC by the cystoscopic view at a follow-up of 18 months (**Figure 1**). One case of urethral stricture was resolved by regular urethral dilation in 2 months. There were two cases of retrograde ejaculation.

**Table 1:** Baseline characteristics and perioperative and follow-up results of patients (*n* = 53).

Parameter	Mild BNC (>5 mm) (*n* = 24)	Moderate BNC (2–5 mm) (*n* = 19)	Severe BNC (<2 mm) (*n* = 10)
Age (year)	74.96 ± 8.01	74.06 ± 7.08	69.0 (65.75, 78.50)
Previous prostate volume (mL)	32.49 ± 10.52	32.86 ± 8.06	29.20 ± 6.69
Occurrence time of BNC (month)	7.50 (3.00, 33.00)	11.00 (4.75, 39.00)	9.50 (4.75, 24.00)
Previous surgical procedure			
TURP	15	8	5
PVP	7	8	4
Other	2	3	1
IPSS	26.46 ± 4.85	28.44 ± 5.10	29.30 ± 4.69
PVR (mL)	71.00 (48.00, 93.00)	124.00 (65.00, 227.75)	234.60 ± 189.11
Qmax (mL/s)	6.73 ± 3.29	4.72 ± 3.70	2.26 ± 1.87
Operative time (min)	32.67 ± 11.50	39.67 ± 14.99	43.00 ± 18.59
Bladder irrigation time (h)	17.88 ± 5.61	17.50 (13.50, 20.00)	20.50 (15.00, 32.00)
Postoperative hospital stay (day)	3.50 (3.00, 5.00)	3.00 (2.75, 5.00)	4.40 ± 1.96
Excised scar tissue (g)	1.25 (0.35, 4.73)	2.85 (0.73, 11.05)	8.06 ± 4.35
Complications			
Blood loss (mL)	5.00 (5.00, 13.75)	10.00 (10.00, 15.00)	10.00 (5.00, 21.25)
Dysuria	0	1	1
Incontinence	0	0	0
Urethral stricture	1	0	0
Retrograde ejaculation	1	1	0
BNC recurrence	3	2	0

All cases that underwent follow-up assessment (**Table 2**) at 3 and 6 months after the surgery showed significantly decreased IPSS and PVR and increased Qmax compared to preoperation (*p* ≤ 0.001). All cases that underwent follow-up assessment 3 months after the surgery showed no significantly decreased IPSS (*p* = 0.331) and PVR (*p* = 0.297) and increased Qmax (*p* = 0.373) compared to 6-month data. Of these 53 patients, there was no recurrence of BNC in severe BNC patients, but a total 5 of 53 (9.4%) BNC patients developed BNC again within 6 months and required repeat TUIBN. Thirty patients comprised five recurrent cases with a follow-up period of more than 1 year and five patients with a follow-up period of more than 2 years, and there was no recurrence of BNC. However, routine and periodic cystoscopy for each patient is not ethical and is unnecessary in clinical practice. So, only two patients followed by the view of cystoscopy. An example of a severe BNC patient, whose bladder neck contracted to a pinpoint-like hole preoperatively and obtained a persistent wide bladder neck at 18 months after TUIBN, is presented in **Figure 1**. According to the modified Clavien–Dindo classification, there are two postoperative complications to patients, which were graded as grade II. The rest of the postoperative complications to patients were graded as grade I.

**Table 2:** Data at baseline and 3- and 6-month after surgery.

Parameter	Baseline (*n* = 53)	3-Month (*n* = 53)	6-Month (*n* = 53)	*F*	*p* ^a^	*F*	*p* ^b^	*F*	*p* ^c^
IPSS	28.00 (23.00, 32.00)	15.00 (10.00, 21.00)	15.00 (10.00, 25.00)	Z = 5.848	<0.001	Z = 0.972	0.331	Z = 3.399	0.001
PVR	86.00 (63.50, 179.00)	5.00 (3.00, 6.00)	4.00 (3.00, 5.50)	Z = 6.334	<0.001	Z = 1.043	0.297	Z = 6.334	<0.001
Qmax	4.90 (2.20, 7.85)	20.75 ± 4.93	21.46 ± 5.07	t = 19.852	<0.001	t = 0.898	0.373	t = 21.400	<0.001

*The normal distribution data were represented by mean ± standard deviation, and non-normal distribution data were represented by median and quartile. The comparison of indexes before and after surgery was self-paired (self-paired design, using paired t-test or paired rank-sum test according to whether the difference was normally distributed), Z was the Z-value of paired rank-sum test, T was the T-value of paired t-test, and p < 0.05 was statistically significant.*

^a^ *3-Month compared with baseline data.*

^b^ *6-Month compared with 3-month data.*

^c^ *6-Month compared with baseline data.*

## Discussion

BNC is a relatively severe complication after endoscopic surgery for BPH. It usually develops within 60 days after transurethral resection (15). Lee (13) reported that the mean time to diagnose BNC after surgery was 18.5 months; however, half of patients developed BNC within in 6 months. If there is no significant improvement in dysuria after urethral dilation, a necessary cystoscopy can confirm the diagnosis due to the possibility of BNC. So far, the exact mechanisms of BNC after TURP remain unclear, and the proposed predisposing factors have been stated as extensive resection of the bladder neck, excessive fulguration at the bladder neck, or a large resecting loop that generates excessive heat to produce a hypertrophic scar in a small intraurethral adenoma (16). Treatment options for BNC remain controversial; urethral dilation 18F followed by a 3-month period of intermittent self-catheterization, cold knife incision, and transurethral resection have been proposed with varying success rates. Regular long-term urethral dilation contributes to a false urethral pathway, hemorrhage, infection, and poor quality of life. About 90% of patients may have to repeat urethral dilation within the first 2 years (17). Eltahawy et al. demonstrated a success rate of 83% after stricture site irrigation with triamcinolone following BNC ablation with a holmium laser (18). Redshaw et al. showed a success rate of 75% after mitomycin C (MMC) injection following a radial cold-knife incision of BNC. However, for MMC utilization, adverse events were described, such as anaphylaxis, extravasation, or bladder neck necrosis, that need to be noticed (19).

Patients with refractory BNC are advocated for more invasive procedures such as YV-plasty, TV-plasty, T-plasty (a modified YV-plasty) (11), and even robot-assisted laparoscopic Y-V plasty (RAYV) (12). The analysis of subjective satisfaction showed that patient satisfaction was very high, high, and undecided in 70%, 20%, and 10%, respectively, with the T-plasty procedure; however, the mean time of operation was 112 min, and the mean hospital stay was 13 days. Musch et al. presented a success rate of RAYV of more than 80%, with a median follow-up of 23.2 months. However, the surgical time ranged from 140 to 360 min, and the blood loss was more than 50 mL. Therefore, these complicated procedures are not accepted among BNC patients initially but for the refractory BNC.

It appeared that a prophylactic bladder neck incision could protect against the formation of bladder neck contracture and reduce the incidence of BNC (4, 20). According to these results, BNI may be beneficial for the treatment of BNC. Ramirez et al. reported a success rate of 72% at a mean follow-up of 12.9 months after Collings knife (the same as the needle-type electrode) incision of BNC at 3 and 9 O’clock positions, an overall success rate of 86% after two procedures (21). Rosenbaum et al. showed that the success rate after TUIBN at 4, 8, and 12 O’clock positions was 45%, and the risk factors associated with recurrent BNC were >10 pack/year history of smoking and patients who had undergone more than two previous TUIBN procedures (22). In the present study, the incision of BNC was carried out at 5, 7, and 12 O’clock positions, resulting in 48 (90.6%) successful cases.

Our present study showed scar tissue of prostatic urethra (**Figure 2**) formed in patients, which combined with BNC promoted dysuria. That is why we performed the incision not only at the bladder neck but also extended the incision from the bladder neck to the proximal verumontanum. The mechanism of TUIBN for the treatment of BNC may be lowering the pressure by destroying a part of the adrenergic receptors of the prostatic fascia (13). A greater degree of destruction of the sympathetic innervation can be obtained through deep trilateral incisions.

The needle-type electrode has the advantage of precise hemostasis and cutting to avoid urinary extravasation or incontinence. If venous sinus hemorrhage occurs, 1 h of compression by F22 catheterization with 45 mL water injection in a balloon was performed for hemostasis. In the present study, several cases of venous sinus hemorrhage were treated with this method efficiently, and the total blood loss was within 50 mL in each case. For those severe BNC cases, especially patients with false urethral pathways caused by incorrect urethral dilation, it is very difficult to find the right urethral pathway under a resectoscope. So, a guidewire should be used to identify a correct pathway under the ureteroscope through the urethra or bladder (14).

From our point of view, this approach brings more advantages compared to the previous endoscopic incision and reanastomosis for BNC. A short operative time, less blood loss, fewer complications, and a higher success rate (90.6%) were also presented. It is noteworthy that TUIBN seemed more likely to treat the severe BNC without recurrence of BNC in the present study. Specifically, this easy method will benefit the surgeon who holds the essential TURP skills for the treatment of BNC.

Limitations of this study are that it is a retrospective study and a relatively small number of patients for follow-up are included. Thus, the incidence of recurrent BNC could have been underestimated in this study.

## Conclusions

This method was reported on TUIBN at three points with a needle-type electrode for BNC. As performed, the method was feasible for all patients and easy to perform. At the same time, no major intraoperative or postoperative complications were observed. Certainly, more clinical data with a longer follow-up are needed to reveal the actual efficacy and relevance of TUIBN for BNC.

## Data Availability

The original contributions presented in the study are included in the article/supplementary material; further inquiries can be directed to the corresponding author/s.
